# Hepatocellular Carcinoma in Patients With Liver Cirrhosis Secondary to Autoimmune Hepatitis: A Case Series and Literature Review

**DOI:** 10.7759/cureus.30698

**Published:** 2022-10-26

**Authors:** Hassan Perera Mesa, Lisbetty Lugo, Ellery Altshuler

**Affiliations:** 1 College of Medicine, University of Florida Health, Gainesville, USA; 2 Internal Medicine, University of Florida Health, Gainesville, USA; 3 Internal Medicine, University of Florida College of Medicine, Gainesville, USA

**Keywords:** liver transplant, autoimmune hepatitis surveillance, liver cirrhosis, hepatocellular carcinoma screening, autoimmune hepatitis with cirrhosis

## Abstract

Autoimmune hepatitis (AIH) is a known risk factor for the development of hepatocellular carcinoma (HCC). However, the current clinical guidelines do not offer a systematic approach to the surveillance and follow-up of patients with AIH to help with the diagnosis and treatment of HCC in this patient population. In this case series, we describe the clinical presentation and management of eight patients who were diagnosed with HCC secondary to underlying AIH at the University of Florida Health Shands Hospital. Throughout their treatment course, all eight patients were identified to have either histological or radiological evidence of liver cirrhosis. Furthermore, all of them tested negative for chronic viral hepatitis serologies and denied any history of excessive alcohol consumption. The median time interval between AIH diagnosis and HCC development in this cohort was 48 months. All demographic, clinical, and laboratory findings were summarized and compared to the relevant data in the existing literature. Our findings suggest that patients diagnosed with AIH would benefit from liver cirrhosis screening and, if present, they should adhere to a regular HCC surveillance schedule.

## Introduction

Hepatocellular carcinoma (HCC) continues to be associated with a great deal of morbidity and mortality worldwide, and it is ranked as the sixth most common type of cancer despite recent medical and technological advances [1-5]. Most often associated with advanced liver disease, the development of liver cirrhosis remains the main indication for initial screening and surveillance of at-risk populations [3-5]. Given the strong association between the development of liver cirrhosis and the diagnosis of HCC, it is crucial to further investigate the risks associated with less common etiologies of cirrhosis.

Autoimmune hepatitis (AIH) refers to an underlying immune-mediated response in individuals with interface hepatitis on liver biopsy, selective IgG hypergammaglobulinemia, and positive for the presence of autoantibodies [1-3,6-13]. Feared complications of AIH include acute liver failure and end-stage liver cirrhosis, both of which could potentially require transplantation and would not respond to standard treatments with pharmacologic immunosuppression and steroids [3,9]. Though previously explored, the relationship between AIH-induced cirrhosis and HCC transformation has not been sufficiently investigated and true incidence remains unavailable for clinical decision-making [12]. Currently, it is clear that patients presenting with evidence of cirrhosis at the time of AIH diagnosis or progression to cirrhosis during follow-up represent the most at-risk population to develop HCC [5,9-10]. This is further endorsed by prior research that has unequivocally identified cirrhosis as the main precursor to HCC development in over 80-90% of the cases studied [12].

Development of HCC has historically been considered a rare occurrence in patients with underlying AIH [1,9-11], and the scarcity of research on this topic could lead to inconsistent delivery of care by clinicians [14]. Guidelines regarding HCC surveillance of patients with AIH and evidence of liver cirrhosis are controversial and have changed over time. There is existing research indicating no formal surveillance schedule reported as per studies from 2008 and 2010 [1,14], while others refer to regular surveillance every six months, as in a follow-up study in 2013 [9], though not specifying which degree of liver cirrhosis would warrant this kind of routine workup. In a recent systematic review and meta-analysis of the incidence and determinants of HCC in patients with AIH in 2017 [12], it has been observed that the American Association for the Study of Liver Diseases (AASLD) guidelines did not make recommendations for routine surveillance in patients with cirrhosis specifically due to AIH, unlike for those with other forms of cirrhosis [12].

## Materials and methods

Study population

In a retrospective chart review conducted at the University of Florida Health Shands Hospital, a large tertiary medical center, patients with diagnosed AIH who developed advanced HCC were identified. The search parameters included patients aged 18 years or older with the International Classification of Diseases, 10th Revision (ICD-10) codes for HCC (C22.0) and AIH (K75.4) between 2007 and 2017. Data for patients with HCC and no history of AIH were not obtained. The exclusion criteria included patients under the age of 18 years, those with incomplete records, or patients with follow-up periods of less than 24 months. Incomplete records were defined as those lacking information on patient age, date of diagnosis, and other key factors used in the statistical analysis. Eight patients were found to meet all the inclusion and none of the exclusion criteria. Following identification and initial screening of the records, baseline demographics and social history were obtained, including age, sex, race, ethnicity, history of smoking, and alcohol use. In addition, a baseline past medical history of hypertension, cardiovascular disease, and diabetes mellitus for each participant was also documented.

Clinical assessment

In terms of clinical data, we recorded the date of diagnosis of AIH, date of diagnosis of HCC, size, and stage of tumor upon initial diagnosis, treatment modality [transarterial chemoembolization (TACE), Y90, or systemic therapy], and date of death, if applicable. Simultaneously, the time interval between AIH diagnosis and HCC development was calculated and reported. Relevant labs at the time of HCC diagnosis were also noted, including Model for End-Stage Liver Disease (MELD) scores, hepatic panels, prothrombin time/international normalized ratio (PT/INR), and platelet counts. Patient records were reviewed for known complications previously described in the current body of literature, such as hepatic encephalopathy, esophageal varices, and spontaneous bacterial peritonitis.

Literature search strategy

For the literature review component, the PubMed database was searched using a combination of MESH terms related to AIH and HCC. The MESH terms used were “autoimmune hepatitis”, “hepatocellular carcinoma”, “autoimmune hepatitis” AND “hepatocellular carcinoma”. Studies published prior to 2000 were excluded. We also used free text terms to locate similar case series and meta-analyses that explored the connection between AIH and HCC.

Statistical analysis

Statistical analysis for this study included Chi-square tests that were performed only to analyze factors associated with medical decision-making incapacity. A 95% confidence interval (CI) and a p-value of 0.05 were used to determine statistical significance. Statistical analysis was performed with SPSS Statistics 28.0.1.0 (142) (IBM Corp, Armonk, NY).

## Results

Demographic data

A total of 1551 patient charts were identified based on the inclusion criteria of having an ICD-10 diagnosis of HCC, of which nine (0.58%) also carried an ICD-10 diagnosis of AIH. The diagnosis was confirmed by positive antinuclear antibodies (ANA), anti-smooth muscle antibodies (ASMA), and/or anti-liver-kidney microsomal antibodies (anti-LKM1) positivity in each patient. One of the patients was excluded due to incomplete records despite having a formal diagnosis of AIH and HCC. The final group comprised six women and two men as shown in Table 1. Four of the eight patients were Caucasian, two were Hispanic, and two were African American. Six out of the eight patients denied any tobacco use, while one was a former smoker, and one was an active smoker. None of the patients had a positive personal history of alcohol abuse. The median age of patients at the time of diagnosis of AIH was 56.0 years (range: 22-64 years) and that for HCC was 59.5 years (range: 30-68 years). The median time interval between AIH diagnosis and HCC development was 48 months (range: 4-137 months).

**Table 1:** Baseline demographic data of patients with HCC secondary to AIH AIH: autoimmune hepatitis; HCC: hepatocellular carcinoma; Dx: diagnosis

No.	Gender	Race	Age at AIH Dx (years)	Age at HCC Dx (years)	Time from AIH Dx to HCC Dx (months)
1	Female	White	64	66	23
2	Male	White	61	61	4
3	Female	Hispanic/Latino	45	47	29
4	Male	White	57	60	46
5	Female	Black or African American	56	68	137
6	Female	Black or African American	22	30	105
7	Female	Hispanic/Latino	56	59	50
8	Female	White	45	52	96

Clinical data

Six of the eight HCC diagnoses were made via radiographic findings of hepatic masses on CT and/or MRI, except for one case in which both the CT and MRI were unremarkable, though a subsequent PET scan revealed a small lesion that was characterized as HCC. One other patient in the cohort was diagnosed with HCC at the time of the liver transplant, which was performed due to advanced cirrhosis secondary to chronic AIH. Additionally, all the patients underwent serology testing for hepatitis A virus (HAV), HBV, and HCV, which were all negative.

The largest tumor size that was recorded at the time of diagnosis was a 17.5 cm mass, while the smallest lesion was only captured on a PET scan and could not be appropriately measured. A significant portion (62.5%) of the cohort initially presented with symptoms of ascites, with two cases being classified into the moderate-to-severe range. These same individuals also presented with esophageal varices on esophageal endoscopy, though only two cases required treatment. Notably, three patients developed hepatic encephalopathy, and all of them were conservatively treated with lactulose. Other significant complications, such as spontaneous bacterial peritonitis and hepatorenal syndrome, were not reported in any of the cases.

Three of the patients underwent radioembolization (Y90), accounting for 37.5% of the cohort. One of these patients (No. 3) also subsequently underwent a liver transplant a year later. Another two patients underwent TACE with one of them (No. 8) requiring systemic chemotherapy as well due to disease progression as shown in Table 2. One patient (No. 5) underwent systemic therapy only with no Y90 or TACE treatment and two did not receive any therapy since the diagnosis of HCC was established following the liver transplant (No. 1 and 4). Compared to patients with unresectable HCC and other causes of cirrhosis (n=1112), patients with AIH (n=8) were more likely to be female (75% vs. 21%; p<0.001). There was no statistically significant difference in terms of age (55.4 vs. 62.2 years; p=0.076) or baseline MELD (12.3 vs. 11.4; p=0.661). Patients with AIH had longer overall survival (63.6 vs. 31.2 months; p=0.026) and longer progression-free survival (59.9 vs. 29.9 months; p=0.037).

**Table 2:** Individual clinical presentations, treatments, and complications *Unknown tumor size. Lesion only appreciated on PET scan, but not on MRI or CT HCC: hepatocellular carcinoma; HRS: hepatorenal syndrome; TACE: transarterial chemoembolization; PET: positron emission tomography; MRI: magnetic resonance imaging; CT: computed tomography

No.	Initial tumor size (cm)	HCC treatment	Ascites	Ascites severity	Hepatic encephalopathy	Spontaneous bacterial peritonitis	Esophageal varices	Varices treatment	HRS
1	2.3	Transplant	Yes	Slight	No	No	Yes	No	No
2	1.7	Y90	Yes	Moderate-severe	Yes	No	Yes	Yes	No
3	3.7	Y90 and transplant	No	N/A	No	No	No	No	No
4	Unknown*	Transplant	Yes	Moderate-severe	No	No	Yes	Yes	No
5	17.5	Systemic therapy	Yes	Slight	No	No	Yes	No	No
6	10.0	Y90	Yes	Slight	No	No	No	No	No
7	2.4	TACE	Yes	Unknown	Yes	No	Yes	No	No
8	3.8	TACE and systemic therapy	No	N/A	No	No	No	No	No

Lab data

Table 3 shows the data related to the laboratory diagnosis of HCC. All patients showed elevated levels of alpha-fetoprotein (AFP) with mild to extremely elevated values at the time of HCC diagnosis except for one patient (No. 8). Albumin levels were measured for all eight patients and found to be decreased in six as shown in Table 3. Seven of eight patients had elevated bilirubin levels at the time of HCC diagnosis. Similarly, all patients showed clear laboratory findings of elevated liver enzymes except the last patient (No. 8), with a more marked elevation in aspartate aminotransferase (AST) levels across the cohort when compared to alanine aminotransferase (ALT). Six of the eight patients showed elevated PT levels, though only four of these patients presented with accompanying elevated INR levels. In addition, all others showed marked levels of thrombocytopenia except for one patient (No. 5) whose result remained within the normal range, and one patient (No. 4) for whom this value was not available. MELD scores were calculated for the patients at the time of HCC diagnosis and followed throughout their treatment. At presentation, only two of the eight patients had a MELD score of less than 10. MELD score values ranged from 8 to 22 with an average of 13.0 in this cohort. Notably, all patients were screened for HAV, HBV, and HCV for risk stratification, and all were found to have negative serologies.

**Table 3:** Laboratory values measured at the time of HCC diagnosis HCC: hepatocellular carcinoma; NI: normal interval; PT: prothrombin time; INR: international normalized ratio; AST: aspartate aminotransferase; ALT: alanine transaminase; PLT: platelets; AFP: alpha-fetoprotein; MELD: model for end-stage liver disease; HAV: hepatitis A virus; HBV: hepatitis B virus; HCV: hepatitis C virus

No.	Albumin (g/L) [NI: 3.5-5]	PT (U/L) [NI: 11-13.5]	INR (U/L) [NI: 0.8-1.1]	AST (U/L) [NI: 0-37]	ALT (U/L) [NI: 0-41]	PLT [NI: 150-450]	Bilirubin [NI: 0-1]	AFP [NI: 1.6-4.5 ng/mL]	MELD	Viral hepatitis screening (HAV/HBV/HCV)
1	2.8 (L)	18.3 (H)	1.6 (H)	62 (H)	68 (H)	72 (L)	2.8 (H)	12.5 (H)	16	Negative
2	2.7 (L)	14.7 (H)	1.1	130 (H)	114 (H)	127 (L)	1.6 (H)	4.6 (H)	10	Negative
3	3.9	11.5	1.1	55 (H)	30	91 (L)	1.3 (H)	1247 (H)	8	Negative
4	3.6	14.3 (H)	1.1	153 (H)	65 (H)	----	2.8 (H)	50000 (H)	14	Negative
5	2.9 (L)	18.8 (H)	1.6 (H)	128 (H)	15	288	0.6	1316 (H)	10	Negative
6	3 (L)	15.9 (H)	1.4 (H)	145 (H)	243 (H)	103 (L)	5.4 (H)	151.4 (H)	22	Negative
7	3.2 (L)	12.5	1.1	40 (H)	29	100 (L)	1.1 (H)	5.5 (H)	8	Negative
8	3.3 (L)	17.4 (H)	1.5 (H)	37	21	108 (L)	4 (H)	2.0	16	Negative

## Discussion

AIH is an immune-mediated chronic inflammatory state characterized by autoantibodies against liver parenchyma and associated with a diverse phenotypical presentation [2,6,9]. The diagnosis of this condition is multifaceted with histological signs of interface hepatitis with laboratory evidence of elevated serum levels of IgG, AST, and ALT as well as the definitive presence of autoantibodies (ANA, SMA, or anti-LKM1 positivity) [6-10]. HCC is known to be one of the most undesirable complications in the clinical course of AIH [5,12]. Due to a paucity of research studies examining this correlation, the exact incidence rates are uncertain. However, a recent meta-analysis [12] revealed that although the pooled incidence for AIH cirrhosis was 1.007% per year, the 95% CI in five out of their 16 studies did fulfill the recommended cut-off value of 1.5% incidence as per AASLD guidelines to suggest routine screening as cost-effective (according to AASLD guidelines, HCC surveillance is cost-effective if the expected risk of HCC exceeds 1.5% per year).

This study outlines the pathological and clinical progression of eight patients at the University of Florida Health Shands Hospital, all of whom were diagnosed and treated for AIH and were subsequently found to have evidence of HCC. In patients with underlying AIH, radiological or histological evidence of cirrhosis has been established as the main risk factor for the development of HCC [6,9,15], while AIH patients without evidence of cirrhosis are at extremely low risk of HCC progression [14,15]. Similarly, our study revealed a significant association between the development of AIH cirrhosis and the subsequent diagnosis of HCC. This relationship presents yet another layer of complexity, as prior research has found that the duration of cirrhosis is a major factor that increases the risk of HCC in patients with AIH [6,9]. In one particular study aimed at evaluating the rate of HCC development in patients with AIH [1], the median duration of liver cirrhosis prior to developing HCC was 102 months, with a range of 12-195 months. Although our study did not specifically look at the interval between formal diagnosis of cirrhosis and HCC diagnosis, all eight patients examined did have histological and/or radiological evidence of cirrhosis following their AIH diagnosis, but prior to their HCC diagnosis. Moreover, our study revealed a mean interval of 48 months between the respective AIH and HCC diagnoses for all eight patients. Surprisingly, this was a significantly shorter interval than what was previously described in the literature, and further research would be beneficial in exploring the underlying factors to explain this finding. If such a faster progression were to be clinically present, guideline changes would provide better clinical guidance and could lead to better prognoses and overall survival rates.

Other predictive risk factors include the manifestation of esophageal varices, ascites, and thrombocytopenia, as described in previous research [6]. The majority of the patients in this study had a well-documented history of varices and/or ascites, and all but one (No. 4) showed laboratory evidence of thrombocytopenia. Although a clear relationship has not been established, AIH has occasionally been linked to underlying serological evidence of viral infections such as HCV [8]. Though often considered a diagnosis of exclusion, AIH has been concurrently reported in patients with viral hepatitis [11,13]. Notably, all the patients in our study were screened for and showed no evidence of previously undetected viral hepatitis, eliminating this as a possible etiology or confounding factor in assessing the association between AIH pathological risk of HCC development.

## Conclusions

The development of liver cirrhosis remains the primary indicator and risk factor for the development of HCC, especially in patients with underlying AIH. Due to the chronic inflammatory state and fibrosis, AIH should be definitely accounted for in risk stratification for patients with an increased likelihood of disease progression. Though no current guidelines exist to assist clinicians, our study suggests that surveillance for liver cirrhosis should be prioritized in patients with a diagnosis of AIH, which further supports findings from prior studies. When compared to viral hepatitis, AIH should be perceived as the initial insult that spurs the development of liver cirrhosis. If missed, this advance would put patients at risk of HCC even in the absence of overt clinical symptoms. Moreover, due to the relatively short intervals between the respective AIH and HCC diagnoses, our findings suggest that patients with AIH and evidence of liver cirrhosis should follow a strict HCC surveillance schedule for earlier detection and treatment of possible malignancies.
